# Health outcomes, education, healthcare delivery and quality – 3045: Questionnaire survey of patients with allergic disorders in Bangalore, India

**DOI:** 10.1186/1939-4551-6-S1-P216

**Published:** 2013-04-23

**Authors:** Eash Hoskote, Suma Kumar, Narasimhan Rajgopal

**Affiliations:** 1 Pediatrics, Excelcare Hospital, Bangalore, India; 2 Allergy Asthma and Chest Centre, India

## Background

Allergic Disorders are on the rise worldwide and especially in Indian urban set up. Bangalore is an urban city with rising population density and pollution. We evaluated the common symptoms of various allergic disorders in three selected outpatient clinics through a questionnaire survey, in children and adults who presented with symptoms to suggest allergy especially rhinitis and asthma.

## Methods

Internal consistency of the questionnaire was checked. Eighty three patients with symptoms to suggest allergy participated in the survey. We evaluated the common symptoms, duration, family history, perceived triggers and treatment sought through the questionnaire survey. A proportion of them (12%) had skin prick tests (HollisterStier, USA) to correlate between perceived and the actual triggers.

## Results

Forty six patients (55.4%) were males and 44.6% (n=37) females. Common Symptoms in children ≤ 12 yrs (n=47, 56.6% of the group), included runny nose (49%), sneezing, nasal obstruction (46.8%) and cough (72.3%). In adults, sneezing and cough predominated equally (63.8%) along with runny nose (50%). Allergic Rhinitis with Asthma was equally seen in adults (22%) and children (19%). 45.8% of our study patients had family history of allergic disorders. Symptoms lasted > 3 to 4 days in a week in the majority (65 %). Commonest perceived triggers included dust (77%), strong odours (24.1%), food items both in children and adults. Discrepancy in perceived and identified triggers by skin prick testing was evident in 2 (out of 10) subjects. 13% of the group was on alternative medicine (ayurveda, homeopathy) at the time of the study and 10 patients (12%) were using only reliever inhalers in spite of ongoing symptoms to suggest asthma.

## Conclusions

Coexistence of Allergic Rhinitis and Asthma was equally common in adults and children. Dust exposure was the commonest trigger and the differences in perceived and identified triggers should be tested in larger patient groups. As depicted in other studies, suboptimal control of allergic disorders was prevalent in our study patients as well.

